# Impact of alcohol consumption on outcomes and potential of immune biomarkers for postoperative complications in trauma patients

**DOI:** 10.3389/fimmu.2025.1492288

**Published:** 2025-04-14

**Authors:** Mohammad Majd Hammour, Yelda Anuk, Regina Breinbauer, Romina H. Aspera-Werz, Yuxuan Xin, Guanqiao Chen, Tina Histing, Sabrina Ehnert, Andreas K. Nüssler, Stefan Döbele

**Affiliations:** ^1^ Department of Traumatology, Siegfried Weller Institute, BG-Clinic Tübingen, Eberhard Karls University, Tübingen, Germany; ^2^ Department Traumatology and Reconstructive Surgery BG-Clinic Tübingen, Tübingen, Germany

**Keywords:** trauma, alcohol, postoperative complications, clinical outcomes, biomarkers

## Abstract

**Introduction:**

Alcohol consumption is a significant risk factor for adverse outcomes in trauma patients. Despite this, effective predictive biomarkers for postoperative complications remain elusive. This study aims to identify potential immune system biomarkers associated with postoperative complications in trauma patients with a history of chronic alcohol consumption.

**Methods:**

A prospective cohort study was conducted on trauma patients admitted to a level 1 Trauma Center. Chronic alcohol consumption and drinking habits were assessed using the Alcohol Use Disorders Identification Test (AUDIT-C) questionnaire. Specifically, 26% of patients reported no alcohol consumption, 44% reported moderate alcohol consumption, and 30% were identified as having risky alcohol consumption. Acute systemic alcohol levels at the time of injury were not measured or considered in this study, as the focus was on chronic consumption patterns. Routine blood screening data were analyzed.

**Results:**

Except for CRP, blood values were comparable between patients with risky alcohol consumption and controls. However, CRP’s ability to predict complications in patients with risky alcohol consumption remained limited (ROC-AUC = 0.6288). In order to identify other predictive markers, patients were matched based on relevant covariates in further analyses. Cytokine Array screening identified CD28, B7-1, Eotaxin-3, TIMP-1, and IL-13 as potential markers to predict complications. Verification with ELISA, however, showed that potential differences could only be detected in the control group. The discrepancies observed between cytokine array and ELISA results can be best explained by methodological differences, particularly since the serum samples were pooled for initial target screening. Additionally, variations in assay sensitivity, dynamic range, and calibration protocols contribute to these discrepancies.

**Discussion:**

These findings suggest that chronic alcohol consumption alters cytokine responses, posing challenges for identifying reliable immune biomarkers for postoperative complications. Future studies should explore alternative approaches for biomarker validation and consider individualized assessment strategies for trauma patients with alcohol consumption history.

## 1 Introduction

Alcohol consumption is a major public health concern worldwide, with significant consequences for individuals and societies (1). It is linked to a wide spectrum of diseases, such as alcohol dependency, liver diseases, cardiovascular diseases, cancer, and impaired bone homeostasis (2, 3). However, alcohol remains deeply integrated into the social behavior in many cultures (4). According to the demographical report from the Robert Koch Institute in Berlin, 13.1% of women and 18.5% of men in Germany consume alcohol in harmful quantities (5). This places Germany among the top alcohol consumers in Europe, with an average of 10.6 liters *per capita* (3). Moreover, alcohol consumption not only causes numerous chronic diseases but also increases the risk of accidents and injuries (6). In trauma settings, many studies have focused on the effects of acute alcohol intoxication, which is known to increase the risk of injury by impairing judgment and coordination. It also significantly influenced the severity of injuries or the long-term outcomes, such as delayed wound healing, prolonged hospital stays, and higher rates of complications (6, 7). While acute intoxication is undoubtedly essential, our study focuses on the effects of chronic alcohol abuse. Chronic alcohol abuse can result in immunosuppression, increasing the risk of infections and delaying wound healing (8–10). Despite the established association between alcohol consumption and adverse outcomes in trauma patients, alcohol intake alone cannot reliably predict complications. The amount of alcohol intake, especially when measured as an acute value, does not consistently correlate with the severity of complications, indicating that acute alcohol consumption by itself is not a sufficient predictor (11). Therefore, there remains a need for reliable biomarkers that reflect the cumulative impact of long-term alcohol consumption and could predict which patients are at higher risk for complications. Identifying such markers may enable clinicians to provide more targeted interventions and improve outcome prognosis for their patients.

The chronic impact of chronic alcohol consumption extends beyond direct tissue damage; it is also reflected in metabolic and nutritional biomarkers. Previous studies have explored the relationship between potential pre-operative biomarkers and postoperative complications. For instance, one of the studies showed that elevated pre-operative Hemoglobin A1c (HbA1c) levels could predict complications such as infections and increased mortality in non-diabetic patients (12, 13). Additionally, nutritional biomarkers like serum pre-albumin, transferrin, or insulin-like growth factor 1 (IGF1) have been studied for their potential to predict complications in patients undergoing elective abdominal surgery (14). C-reactive Protein (CRP) kinetics have also been examined in elderly patients with hip fractures, showing an association between elevated CRP levels and complications like infections and delirium (15, 16). However, these studies face several limitations. Many had small sample sizes and observational designs, limiting the generalizability of their findings. Many of these biomarkers have not been validated to determine whether they can predict complications in patients with alcohol consumption. Additionally, confounding factors such as smoking are not always controlled, potentially influencing the clinical outcomes (17). Inconsistencies in the methods of biomarker measurement also pose serious challenges.

In this study, the complex relationship between alcohol consumption, trauma clinic outcomes, and the pursuit of predictive biomarkers is being explored. Drawing on data collected from a cohort of trauma patients, we explored the association between alcohol consumption and the incidence of postoperative complications. Furthermore, we present findings from our analyses of blood samples, utilizing cytokine arrays and Enzyme-linked immunosorbent assays (ELISA) assays to identify potential immune parameters that may serve as early indicators of postoperative complications in trauma patients with a history of alcohol consumption. By shedding light on these critical issues, this paper aims to contribute to the growing body of knowledge surrounding alcohol-related trauma outcomes. Further, it provides new insights that can inform clinical practice and interventions aimed at mitigating the burden of trauma in populations with a propensity for alcohol consumption.

## 2 Materials and methods

### 2.1 Ethical approval

This study was conducted following the Declaration of Helsinki (1964) and was approved by ethical vote 346/2015B02 of the ethical commission of the medical faculty of the University of Tübingen. All participants obtained written informed consent before inclusion in the study.

### 2.2 Study population, inclusion criteria, and data collection

This study included trauma patients admitted to a level 1 trauma center between July 2020 and August 2022. Included were patients aged 18 years or older who experienced a trauma injury requiring surgical intervention or undergone elective surgery and who consented to participate in the study. Patients from the trauma, septic, and endoprosthesis departments were included. Elective procedures in the endoprosthetics department, such as joint replacements and surgeries for non-trauma-related conditions, were not excluded from the analysis as they contribute to the cohort’s full representation of patient outcomes. The patients included in the septic department did not have septic conditions prior to trauma, and only those without sepsis were considered in the final dataset. Included patients self-reported their alcohol consumption using the Alcohol Use Disorders Identification Test (AUDIT-C) questionnaire. Demographic and clinical data were collected from electronic medical records, including age, gender, body mass index (BMI), laboratory data, and postoperative outcomes. Patients were categorized based on the injury site and type of surgical intervention, as detailed trauma severity scores (e.g., Injury Severity Score, ISS) were unavailable for this cohort. Excluded were patients aged under 18 years, polytrauma cases, patients with cognitive impairments or insufficient language skills, those who did not complete the 3-month follow-up, and Patients with septic conditions at the time of inclusion. Additionally, elective patients from the arthroplasty department were included in the analysis to represent the full spectrum of outcomes. Postoperative complications were classified based on their clinical characteristics into implant-associated and non-implant-associated infections, surgery-related complications, internal complications, and preexisting conditions. Additional categories included complications directly related to the surgical procedure, those arising due to the severity of the underlying diagnosis, internal medical complications, and general postoperative complications. Preexisting conditions and other reasons for exclusion were also documented but not considered in the outcome analysis. For this study, implant-associated and non-implant-associated infections, as well as internal and postoperative complications, were included in the final analysis as relevant postoperative complications. The general postoperative complications included wound healing disorders, infections, chronic pain, nerve lesions, movement restrictions, osteoarthritis, hematomas, osteosynthesis failure, and pseudarthrosis.

### 2.3 Blood sampling

Blood samples were collected 1–4 days preoperatively and processed after 30 minutes of collection. Samples were centrifuged for 10 minutes at 4°C at 1000x *g* to obtain blood serum. The serum samples were then aliquoted and stored at -80°C until analysis.

### 2.4 Cytokine arrays

Serum cytokine levels were semi-quantified using Human Cytokine Array C5 and Human Immune Checkpoint Array C1 (RayBiotech, Peachtree Corners, Georgia). The Human Cytokine Array C5 is a membrane-based antibody array designed to detect and compare the expression levels of 80 human cytokines. The Human Immune Checkpoint Array C1 is another membrane-based array that detects 23 human molecules expressed in T/B cells and antigen-presenting cells. To enhance the detection of group-level differences and reduce individual variability, serum samples from patients within each defined group were pooled prior to analysis. This pooling strategy allowed for obtaining a sufficient volume for robust array experiments and provided an averaged expression profile representative of each group. Cytokines and proteins were semi-quantified using these arrays to detect differently expressed markers across the groups. To eliminate the effect of the confounding factors, patients were matched based on gender, age, BMI, and smoking status. The assay was performed according to the manufacturer’s instructions. Briefly, after incubating the membrane in the blocking buffer for 30 minutes at room temperature, the sample incubation was done overnight at 4°C. Chemiluminescence detection was done upon the incubation with biotinylated antibody cocktail and HRP-streptavidin using a charge-coupled device camera (INTAS Science Imaging, Göttingen, Germany). The signal was quantified using ImageJ software (Version 1.54f, NIH, Bethesda, MD, USA) the data were normalized either to the mean value of the cytokine level or the z-score was calculated using the equation:


$$z=\frac{x-\mu }{\sigma }$$

Where z is the z-score, x is the value, µ is the mean of all samples for the target protein, and σ is the standard deviation of all samples for the target protein.

### 2.5 Enzyme-linked immunosorbent assays

ELISA assays were conducted on the same serum samples that had previously been used for the cytokine arrays. However, because of the limited availability of serum, only a smaller subset of patients could be analyzed. To ensure that the groups were well stratified and comparable, logistic regression analyses were performed to test and control for potential confounding factors, including age, body mass index (BMI), smoking status, comorbidities, and education level. ELISA kits for CD28 (#DY342-05), B7-1 (#DY140) (R&D Systems, Minneapolis, MN, USA), Interleukin 13 (IL-13; #900-K23), Eotaxin-3 (#900-K167), and Tissue Inhibitor of Metalloproteinase 1 (TIMP-1; #900-K438) (PeproTech, Hamburg, Germany) were used according to the manufacturer’s protocols. Absorbance was measured using an Omega Plate Reader (BMG Labtech, Ortenberg, Germany), and concentrations were calculated from standard curves generated with known concentrations of the respective cytokines.

### 2.6 Statistical analysis

Statistical analysis was performed using SAS (Version 16, North Carolina, USA) and GraphPad Prism (Version 8.07, San Diego, USA). Continuous variables were presented as Tukey’s boxplots of two technical replicates (n = 2). The number of patients (N) is specified in the figure legends. Statistical significance was assessed using a two-way ANOVA followed by Tukey’s multiple comparison test. The Mann-Whitney U test was used for comparisons between two groups. Categorical variables were expressed as percentages. Statistical significance was indicated as p < 0.05 (*), p < 0.01 (**), p < 0.001 (***), and p < 0.0001 (****). Logistic regression analysis was used to identify independent predictors of postoperative complications. A p-value < 0.05 was considered statistically significant.

## 3 Results

### 3.1 Alcohol consumption and complications

**Figure 1:**
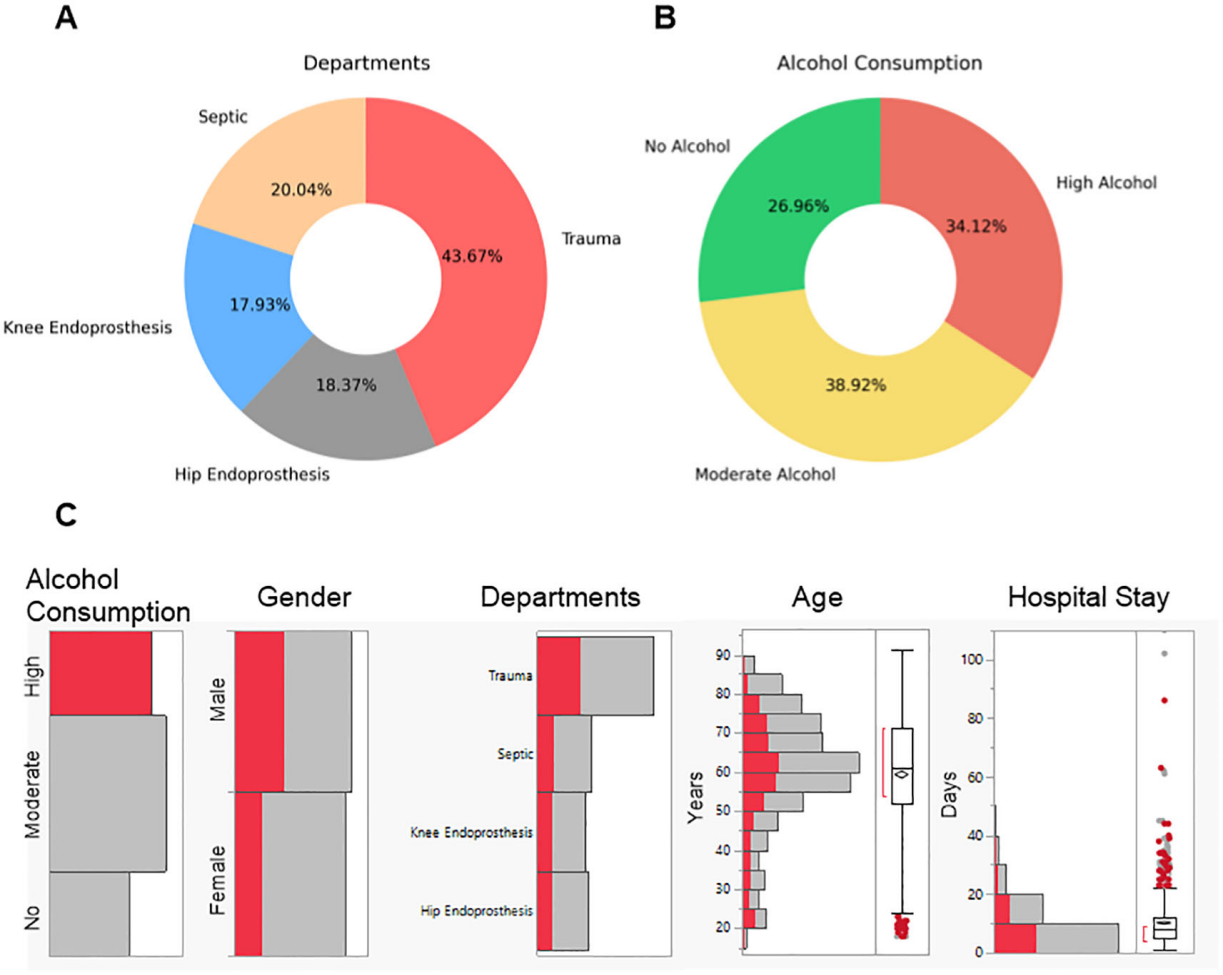
Overview of the patient recruited at a level 1 trauma center for this study. Patient data was collected between 2020 and 2022. **(A)** Distribution of patients in the different departments in the clinic. **(B)** Distribution of alcohol consumption classifications among the patients. No Alcohol represents patients with an AUDIT-C score of 0. Moderate Alcohol represents patients with AUDIT-C scores for females < 3 and males < 4. High Alcohol represents patients with AUDIT-C scores for females ≥ 3 and males ≥ 4. **(C)** Distribution of patients with high alcohol consumption by gender, department, age, and hospital stay duration. The red bars indicate high alcohol consumption, while the gray bars represent moderate or no alcohol consumption.

Between July 2020 and August 2022, 1146 patients were recruited at a level 1 trauma center for this study. These patients were distributed across various departments within the clinic, as illustrated in **Figure 1A**. Specifically, 44% of the patients were treated in the trauma department, 20% in the septic department, and 36% in the endoprosthesis department. Alcohol consumption among the patients was assessed using the AUDIT-C questionnaire. A score of ≥ 3 for females and ≥ 4 for males was used to identify patients with high alcohol consumption, classified as positive for AUDIT-C (18). Based on this classification, 27% of the patients reported no alcohol consumption, 39% consumed alcohol at levels not considered harmful, and 34% reported alcohol consumption at levels considered risky, thus falling into the positive AUDIT-C category, as shown in **Figure 1B**. To explore potential associations, we analyzed alcohol consumption in relation to gender, department, age, and hospital stay duration (**Figure 1C**). A higher proportion of males reported high alcohol consumption, whereas females were more represented in the no-alcohol group. Across departments, alcohol consumption was most prevalent among trauma patients. Age distribution analysis showed that younger patients were more likely to consume alcohol, whereas older patients tended to report lower or no alcohol consumption.

### 3.2 Study population

**Figure 2:**
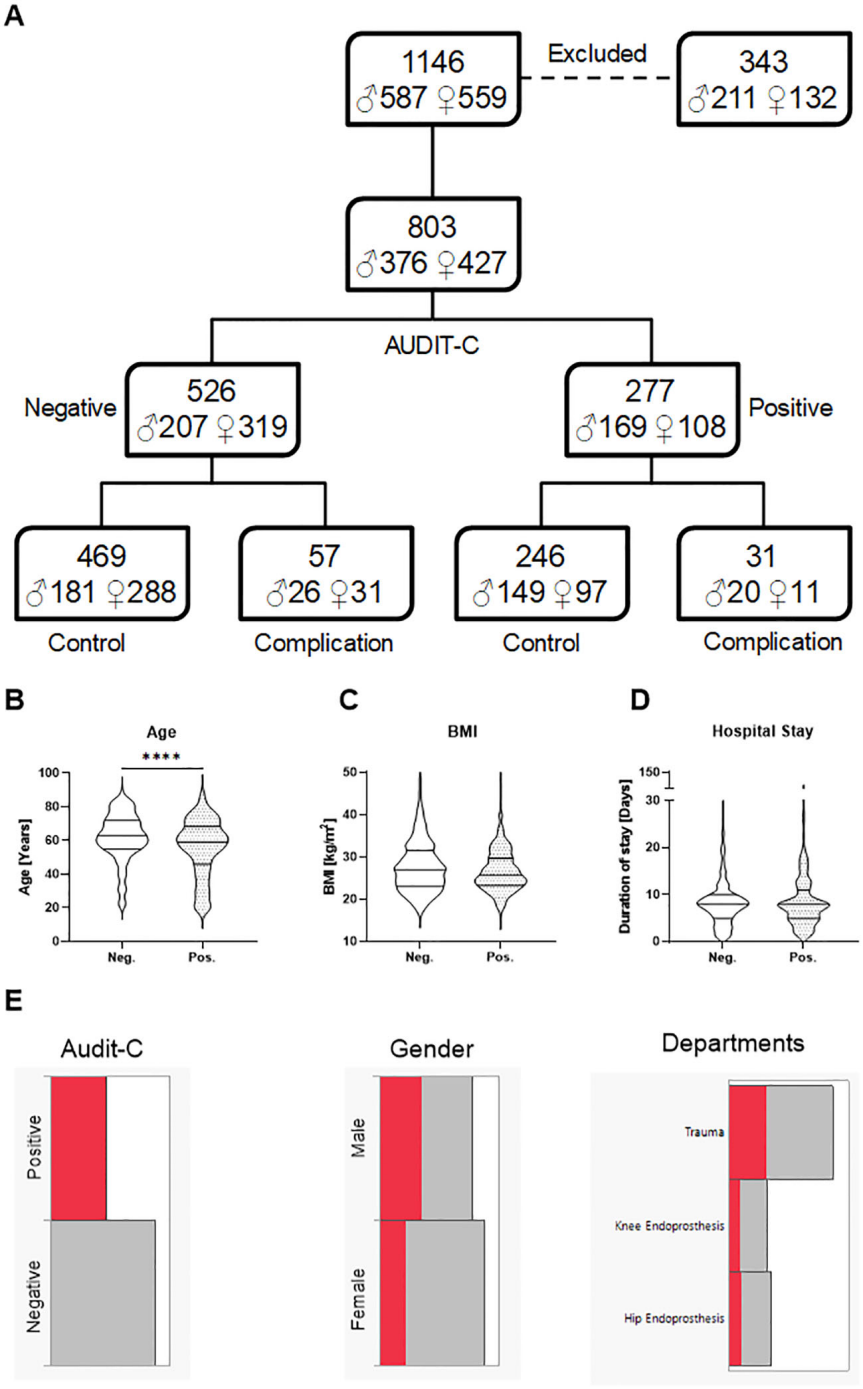
Overview of the study population and comparison of demographic characteristics between patients negative for alcohol risk (Neg.) and positive for alcohol risk (Pos.). Data were collected from 1146 randomly chosen patients in a level 1 trauma center between 2020 and 2022 during their hospitalization time. **(A)** The flow diagram represents the study population: 343 patients were excluded from the study due to pre-existing or procedure-related complications or revision surgery. The patients were divided into two groups depending on their alcohol consumption (AUDIT-C questionnaire) and the classification of the National Institute for Health. 277 patients were classified as positive for alcohol risk (Male: AUDIT-C score ≥ 4, Female: AUDIT-C score ≥ 3) and 526 patients as negative for alcohol risk. The symbol ♂ refers to Male, and ♀ to Female. **(B)** Age, **(C)** Body Mass Index (BMI), and **(D)** duration of hospital stay of the study participants. **(E)** Distribution of patients who are positive for alcohol risk by gender and departments. The red bars indicate high alcohol consumption, while the gray bars represent moderate or no alcohol consumption. Data are presented as violin plots, the lines indicating the median and quartiles. Statistical significance was assessed using the Mann-Whitney U test, with significance indicated by ****p < 0.0001.

In this study, 343 patients were excluded due to pre-existing or procedure-related complications or revision surgery. The remaining patients were categorized based on their alcohol consumption using the AUDIT-C questionnaire. Among these, 277 patients were classified as positive for alcohol risk (Male: AUDIT-C score ≥ 4, Female: AUDIT-C score ≥ 3) and 526 patients as negative for alcohol risk (18). Each group was further subdivided into control (no complications) and complication groups. An overview of the study population is demonstrated in **Figure 2A**. Key demographic and clinical characteristics, including age, BMI, and duration of hospital stay, were compared between patients classified as negative and positive for alcohol risk. As indicated in **Figure 2B**, the positive alcohol risk group was significantly younger than the negative group, with a median age of 59 years compared to 62 years (p < 0.0001). However, there was no significant difference in BMI between the groups, with median values of 27 kg/m² for the negative group and 26 kg/m² for the positive group (**Figure 2C**). We also observed no significant difference in the duration of hospital stay between the two groups, with both having a median stay of 8 days (**Figure 2D**). **Figure 2E** illustrates the distribution of patients identified as having a positive alcohol risk between genders and across the departments.

Furthermore, patient characteristics, comorbidities, and complications are summarized in **Table 1**. Notably, a higher proportion of smokers was observed in the alcohol-positive risk group than in the other groups. In addition, implant-associated infections occurred more frequently in the alcohol-positive risk subgroup. A supplementary table (**Supplementary Table S1**) provides an overview of the 20 most frequently diagnosed diseases and surgical procedures in the study cohort.

**Table 1:** Patient demographic, characteristics, and outcomes stratified by alcohol risk status.

Variable	Negative for Alcohol Risk (n=526)		Positive for Alcohol Risk (n=277)	
Patient Characteristics	Control (n=469)	Complication (n=57)	Control (n=246)	Complication (n=31)
**Age (years, mean ± SD)**	62.09 ± 14.46	60.35^a^ ± 14.18	55.87^c^ ± 17.26	55.09 ± 15.50
**Sex (M/F)**	181/288	26/31	149/97^c^	20/11^b^
**BMI (kg/m^2^, mean ± SD)**	27.58 ± 6.01	29.33^a^ ± 6.34	26.63^c^ ± 5.03	28.05^d^ ± 4.89
**Smoking Status (n smokers)**	74 (15.77%)	24 (42.10%)^a^	71 (28.86%)	13 (41.93%)
**Operation relative to trauma (days, mean ± SD)**	6.18 ± 6.98	8.343 ± 8.01	6.775 ± 6.61	6.808 ± 4.23
**Hospital stay (days, mean ± SD)**	8.37 ± 5.33	9.54 ± 5.70^a^	8.24 ± 5.50	14.51 ± 15.4^b^
Education				
**No qualification**	45 (9.59%)	1 (1.75%)	20 (8.13%)	1 (3.22%)
**Pre-vocational training**	2 (0.42%)	0 (0%)	0	1 (3.22%)
**Lower secondary certificate**	31 (6.60%)	7 (12.28%)	7 (2.84%)	2 (6.45%)
**Two-year vocational training**	193 (29.63%)	20 (35.08%)	96 (39.02%)	10 (32.25%)
**Three-year vocational training**	75 (16%)	12 (21.05%)	40 (16.26%)	4 (12.90%)
**University of Applied Sciences diploma or similar**	68 (14.5%)	10 (17.54%)	40 (16.26%)	5 (16.12%)
**University diploma or similar**	46 (9.80%)	7 (12.28%)	38 (15.44%)	7 (22.58%)
**Doctoral Degree**	9 (1.91%)	0 (0%)	5 (2.03%)	1 (3.22%)
Comorbidities (n)				
**Hypertension**	115 (24.52%)	23 (40.35%)	51 (20.73%)	7 (22.58%)
**Diabetes**	95 (20.25%)	22 (38.59%)	28 (11.38%)^c^	7 (22.58%)
**Cardiovascular disease**	79 (16.84%)	15 (26.31%)	30 (12.19%)	6 (19.35%)
**Endocrine, Nutritional and metabolic diseases**	111 (23.66%)	20 (35.08%)	42 (17.07%)	6 (19.35%)
**Skeletal muscle and connective tissue diseases**	138 (29.42%)	21 (36.84%)	57 (23.17%)	7 (22.58%)
Complications				
**Implant-associated early infection**	–	15 (26.31%)	–	10 (32.25%)
**Implant-associated late infection**	–	0	–	1 (3.22%)
**Not implant-associated infection**	–	5 (8.77%)	–	1 (3.22%)
**Internal complication**	–	2 (3.51%)	–	2 (6.45%)
**Postoperative complication**	–	35 (61.40%)	–	17 (54.83%)

Statistical comparisons were performed using t-tests for continuous variables and chi-square tests for categorical variables. Superscripts indicate statistical significance (p < 0.05): “a” denotes a significant difference between control and complication within the negative alcohol risk group; “b” denotes a significant difference between control and complication within the positive alcohol risk group; “c” denotes a significant difference between the control groups of the negative and positive risk categories; and “d” denotes a significant difference between the complication groups of the negative and positive risk categories.

### 3.3 Clinical laboratory data analysis

**Figure 3:**
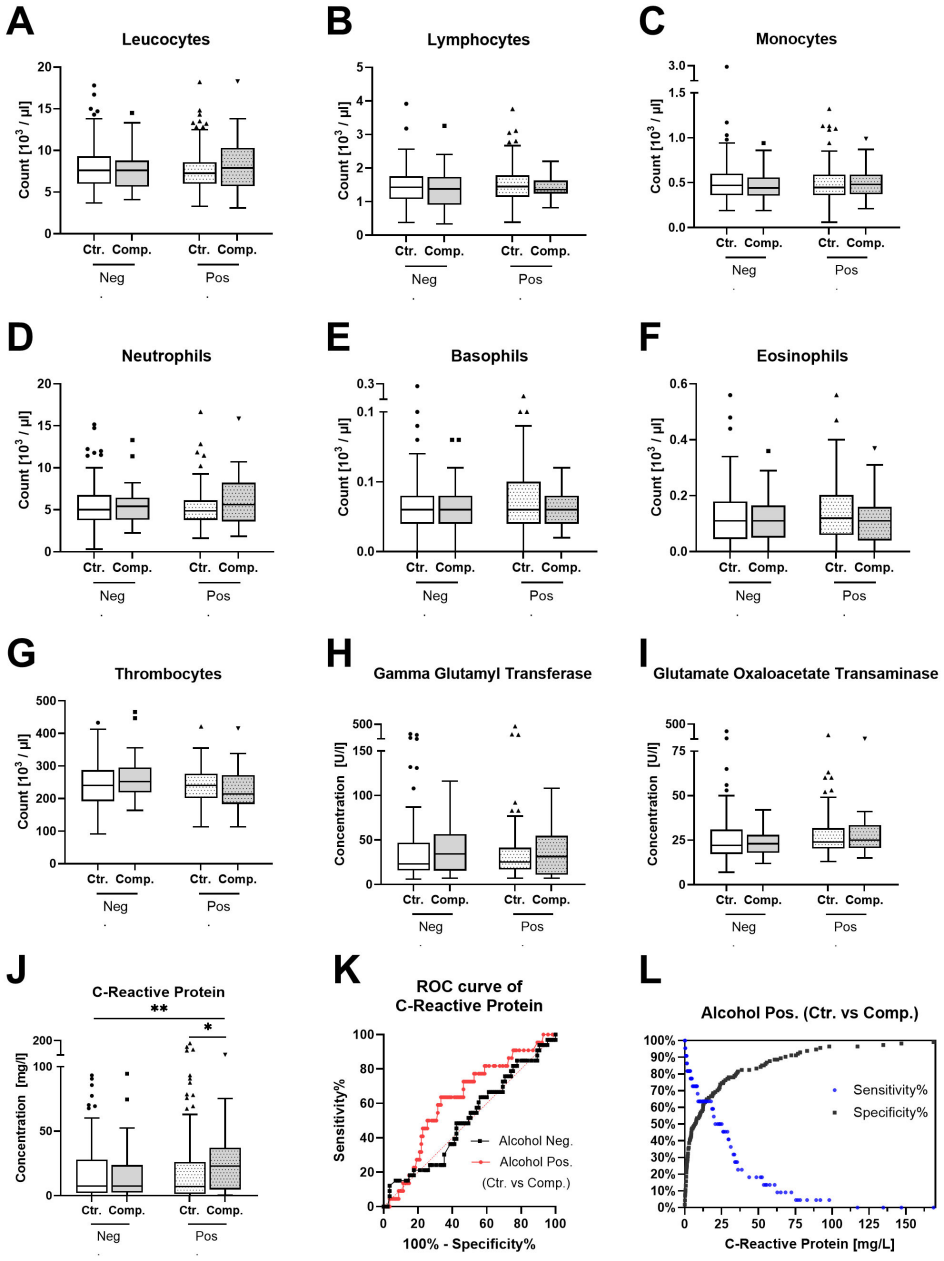
Standard laboratory parameter analysis in patients’ blood samples. Patients were categorized as negative for alcohol risk (Neg.) or positive for alcohol risk (Pos.). Each group is subdivided into a control group (Ctr.) and a complication group (Comp.). The **(A–J)** show the serum levels of **(A)** Leukocytes. **(B)** Lymphocytes. **(C)** Monocytes. **(D)** Neutrophils. **(E)** Basophils. **(F)** Eosinophils. **(G)** Thrombocytes. **(H)** Gamma-glutamyl transferase. **(I)** Glutamate oxaloacetate transaminase. **(J)** C-reactive protein. Data are presented as Tukey’s boxplots of the 4 groups Neg. Ctr. (N=85), Neg. Comp. (N=33), Pos. Ctr. (N=114), and Pos. Comp. (N=23). Statistical significance was assessed using 2-way ANOVA followed by Tukey’s multiple comparisons test, with significance indicated by **p < 0.001. **(K)** Receiver operating characteristic (ROC) curve for CRP in the Neg. and Pos. groups. **(L)** Sensitivity and specificity curve for CRP in alcohol-positive patients, illustrating its performance as a predictive marker for complications.

To identify potential preoperative risk factors for postoperative complications in patients with alcohol abuse, a comprehensive analysis of standard laboratory parameters was conducted. Patients were categorized based on their alcohol risk (Neg. or Pos.) and further subdivided into control (no complications) and complication groups. Logistic regression analyses were performed to control for age, BMI, and smoking status confounding factors. The analyzed parameters included leukocytes, lymphocytes, monocytes, neutrophils, basophils, eosinophils, Thrombocytes, gamma-glutamyl transferase (GGT), glutamate oxaloacetate transaminase (GOT), and CRP (**Figures 3A–J**). Among these, CRP levels in **Figure 3J** showed significantly higher levels in the alcohol-positive complication group compared to both control groups (the main effect of alcohol and complication are significant; p-value = 0,0217, 0,0365, respectively). The Receiver operating characteristic (ROC) curve analysis for CRP within the alcohol-positive group (**Figures 3K, L**) demonstrated a suboptimal discriminatory ability (Area under the curve (AUC) = 0.6288) in predicting complications within this group. **Figure 3L** focuses on alcohol-positive patients, illustrating the sensitivity and specificity of CRP levels for predicting postoperative complications. In contrast, the other parameters showed no significant differences between groups.

### 3.4 Cytokine profile analysis

**Figure 4:**
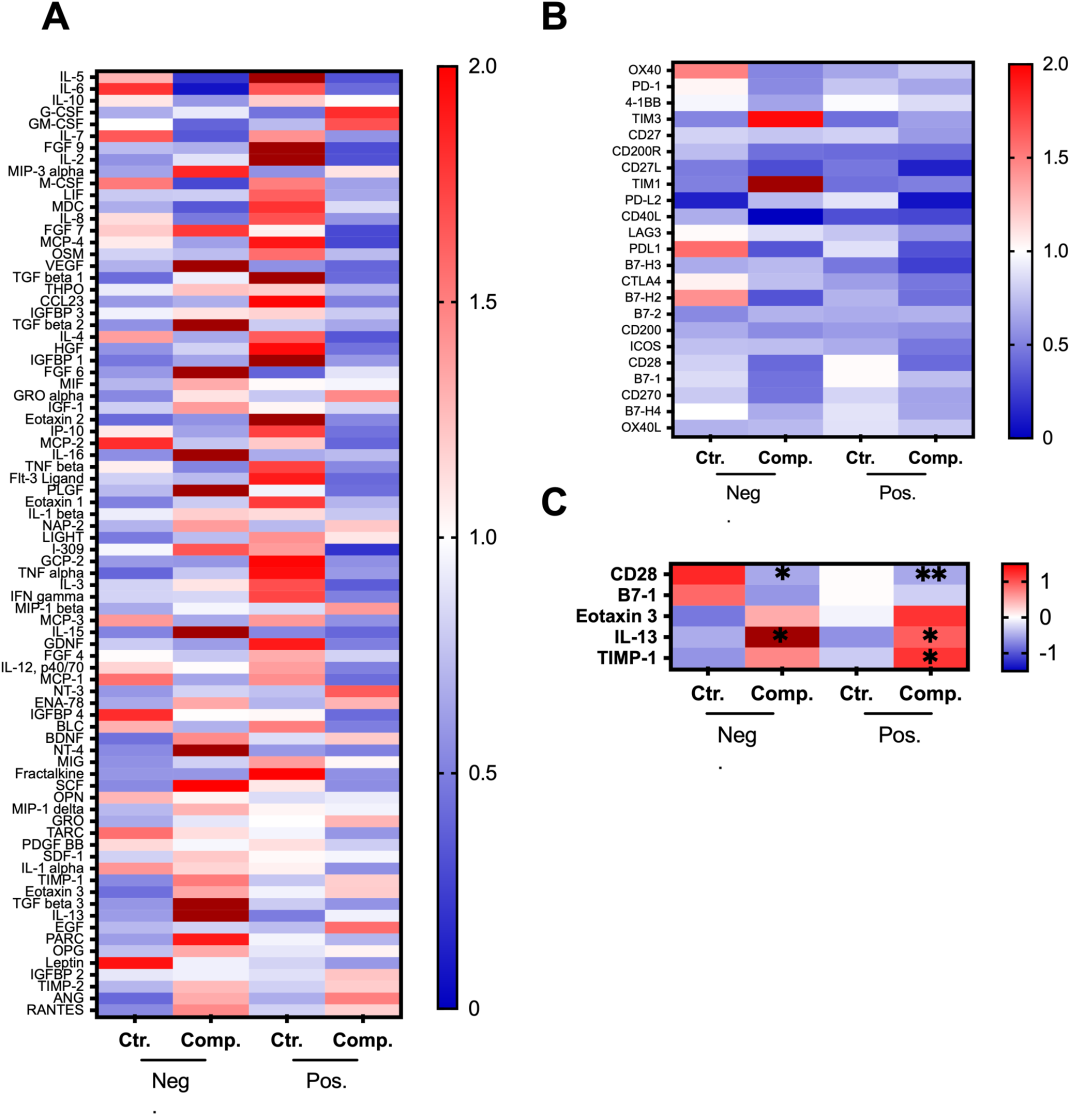
Cytokine array analysis in patient serum samples. Patients were categorized as negative for alcohol risk (Neg.) or positive for alcohol risk (Pos.). Each group is subdivided into a control group (Ctr.) and a complication group (Comp.). The heatmaps represent **(A)** Cytokine levels in the serum samples were determined using Human Cytokine Array C5 from RayBiotech. **(B)** Immune checkpoint protein levels in serum samples were determined using Human Immune Checkpoint Array C1 from RayBiotech. **(C)** Highlights specific cytokines and proteins of interest. The heatmaps represent the cytokine profiles across four matched patient groups, Neg. Ctr. (N=74, pooled), Neg. Comp. (N=74, pooled), Pos. Ctr. (N=22, pooled), and Pos. Comp. (N=22, pooled). The samples of each group were pooled. Data represent 2 rounds with 2 technical replicates for each pooled sample. Data were normalized either to the mean cytokine levels of the groups **(A, B)** or using the z-score **(C)**. The color scale represents the normalized expression levels, with red indicating upregulation and blue indicating downregulation. Differences between groups were assessed using a 2-way ANOVA followed by Tukey’s multiple comparisons test, with significance compared to control in the respective group indicated by *p < 0.05, and **p < 0.001.

A comprehensive screening of cytokines and proteins in blood samples was conducted to identify potential biomarkers for predicting postoperative complications. This screening involved the utilization of the Human Cytokine Array C5 and Human Immune Checkpoint Array C1. The results show that several cytokines displayed variations in expression levels between the groups. To clarify these differences, the data were organized into two panels: **Figure 4A** presents markers predominantly associated with inflammatory responses, while **Figure 4B** focuses on those related to T/B cell activation. This division provided a clearer visualization of group differences. The results show a general trend of increased inflammatory markers in the alcohol-negative complication group and a decrease in the alcohol-positive complication group compared to the controls (**Figure 4A**). In contrast, **Figure 4B** shows a general decrease in the levels of T/B cell activation markers in the complication groups compared to the controls. In **Figure 4C**, specific cytokines of interest were highlighted. This subset of cytokines demonstrated the most pronounced differences. Namely, CD28, B7-1, Eotaxin-3, TIMP-1, and IL-13. These cytokines were also selected due to their relevance to postoperative outcomes. These molecules are involved in T-cell activation, inflammatory response modulation, and tissue remodeling.

### 3.5 ELISA quantification of key markers

**Figure 5:**
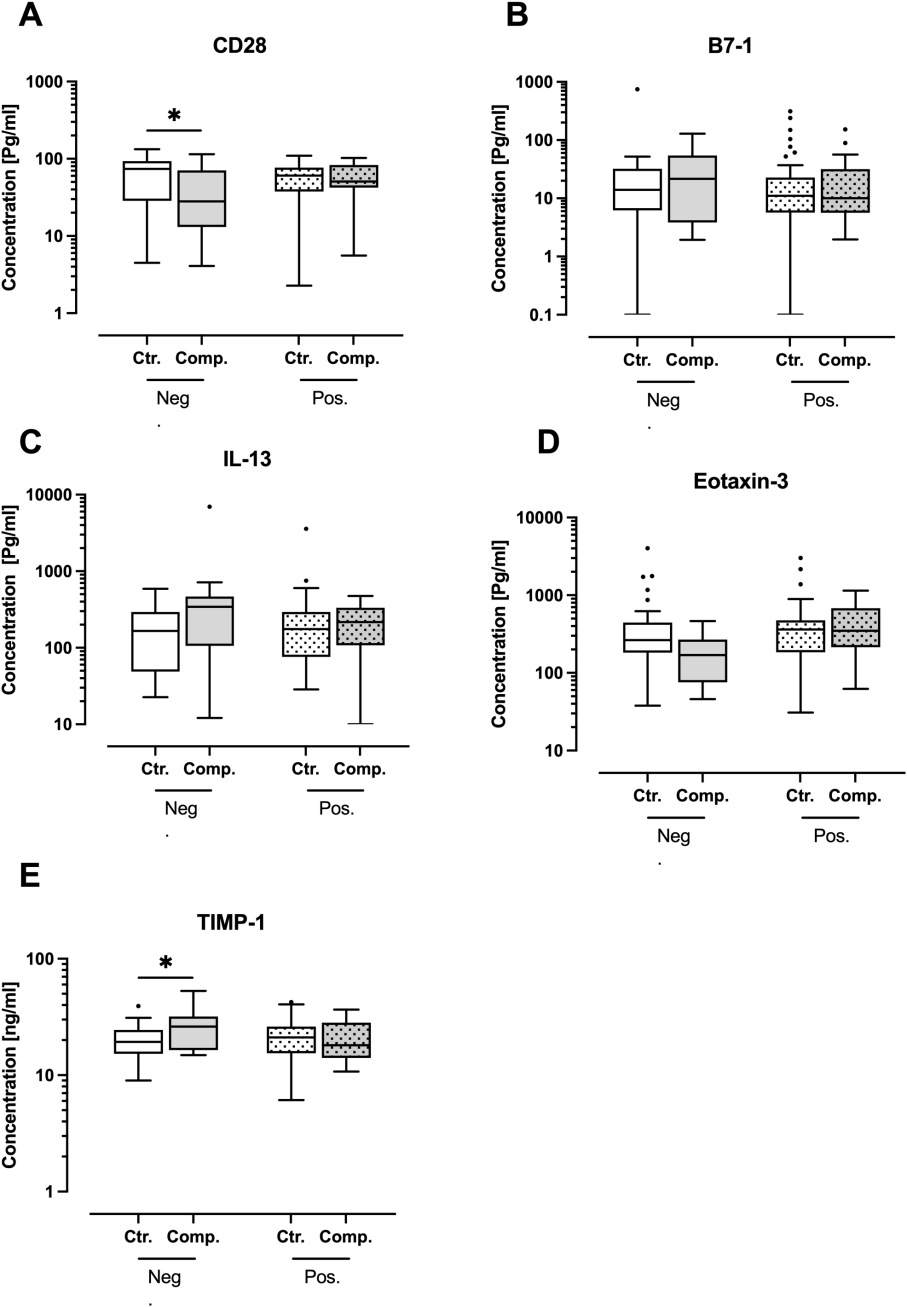
ELISA quantification of key inflammatory and T/B cell activation markers in patient serum samples. Patients were categorized as negative for alcohol risk (Neg.) or positive for alcohol risk (Pos.). Each group is subdivided into a control group (Ctr.) and a complication group (Comp.). The levels of **(A)** CD28, **(B)** B7-1, **(C)** Interleukin 13 (IL-13), **(D)** Eotaxin-3, and **(E)** Tissue Inhibitor of Metalloproteinase 1 (TIMP-1). The data presented as Tukey’s boxplots of the 4 groups Neg. Ctr. (N=31), Neg. Comp. (N=17), Pos. Ctr. (N=46), and Pos. Comp. (N=16). Statistical analysis was performed using 2-way ANOVA followed by Tukey’s multiple comparisons test if the effect of alcohol and/or complication was significant, with significance indicated by *p < 0.05.

The ELISA analysis focused on key cytokines identified as significant in the initial cytokine array analysis. Specifically, CD28, B7-1, Eotaxin-3, TIMP-1, and IL-13. To ensure a balanced comparison, logistic regression analysis was performed and demonstrated no significant differences in baseline characteristics between groups, reinforcing the validity of comparisons. The patient characteristics and comorbidities are summarized in **Table 2**, including percentages for categorical variables. The reduction in sample size for the ELISA analysis was primarily due to limited serum availability. In the alcohol-negative group, there were clear distinctions between the control and complication subgroups. For instance, B7-1, IL-13, and TIMP-1 levels were elevated in patients with complications compared to controls (**Figures 5B, C, E**). The levels of CD28 and Eotaxin-3 were decreased in the complication group (**Figures 5A, D**). However, in the alcohol-positive group, this distinction was not observed. Levels of CD28, B7-1, Eotaxin-3, TIMP-1, and IL-13 (**Figures 5A–E**), remained relatively consistent between the control and complication subgroups.

**Table 2:** Comparison of patient demographics, comorbidities, and postoperative complications between alcohol-negative and alcohol-positive groups included in the ELISA analysis.

Variable	Negative for Alcohol Risk (n=48)		Positive for Alcohol Risk (n=62)		p-value
Patient Characteristics	Control (n=31)	Complication (n=17)	Control (n=46)	Complication (n=16)	(Intercept) 0.896
Age (years, mean ± SD)	57.43 ± 15.73	63.19 ± 11.23	57.31 ± 12.66	59.65 ± 15.78	0.357
Sex (M/F)	15/16	8/9	33/13	9/7	0.593
BMI (kg/m², mean ± SD)	28.75 ± 4.29	28.11 ± 6.29	27.95 ± 4.26	28.24 ± 5.41	0.65
Smoking Status (n smokers)	4 (12.90%)	5 (29.41%)	9 (19.56%)	6 (37.5%)	0.364
Education					
No qualification	1 (3.22%)	0	1 (2.17%)	0	
Pre-vocational training	0	0	0	1 (6.25%)	
Lower secondary certificate	3 (9.67%)	3 (17.64%)	4 (8.69%)	2 (12.5%)	
Two-year vocational training	9 (29.03%)	6 (35.29%)	14 (30.43%)	4 (25%)	
Three-year vocational training	7 (22.58%)	6 (35.29%)	22 (47.82%)	4 (25%)	
University of Applied Sciences diploma or similar	8 (25.80%)	4 (23.52%)	7 (15.21%)	3 (18.75%)	
University diploma or similar	2 (6.45%)	2 (11.76%)	4 (8.69%)	2 (12.5%)	
Doctoral Degree	0	0	0	1 (6.25%)	
Comorbidities (n)					
Hypertension	7 (22.58%)	7 (41.17%)	6 (13.04%)	1 (6.25%)	0.746
Diabetes	2 (6.45%)	3 (17.64%)	11 (23.91%)	6 (37.5%)	0.926
Cardiovascular disease	10 (32.25%)	6 (35.29%)	8 (17.39%)	6 (37.5%)	0.675
Endocrine, Nutritional and metabolic diseases	9 (29.03%)	10 (58.82%)	13 (28.26%)	7 (43.75%)	0.174
Skeletal muscle and connective tissue diseases	16 (51.61%)	4 (23.52%)	28 (60.86%)	5 (31.25%)	0.122
Complications					
Implant-associated early infection	–	5 (29.41%)	–	6 (37.5%)	
Implant-associated late infection	–	0	–	1 (6.25%)	
Not implant-associated infection	–	2 (11.76%)	–	1 (6.25%)	
Internal complication	–	2 (11.76%)	–	2 (12.5%)	
Postoperative complication	–	12 (70.58%)	–	7 (43.75%)	

## 4 Discussion

Alcohol consumption remains a significant public health concern, particularly in clinical settings where patients often present with complications related to their drinking habits (19, 20). In Germany, the prevalence of alcohol consumption is notably high, with approximately 7.8 million adults exhibiting risky alcohol use. Further, around 3.4% of the population meets the criteria for alcohol dependence (21, 22). Within clinical populations, these numbers are even higher, reflecting the impact of alcohol on health (23). Despite the well-established link between alcohol use and adverse health outcomes, there remains a significant need for targeted research specifically examining the impact of alcohol on patients. Previous studies have primarily focused on general populations, lacking the specificity needed to identify biomarkers that reliably predict alcohol-related postoperative complications (24–26). This limitation has hindered the development of targeted strategies for the early detection and prevention of postoperative complications in individuals with high alcohol consumption. Furthermore, while some research has examined the relationship between alcohol and general health outcomes, these studies have not fully clarified how alcohol alters the inflammatory response (27, 28). This gap complicates the identification of reliable biomarkers crucial for anticipating surgical complications related to alcohol use. Addressing these research gaps could significantly enhance patient care and outcomes in surgical settings. In this study, the interplay between alcohol consumption, trauma clinic outcomes, and the search for predictive biomarkers is explored. This relationship is vital for understanding how alcohol influences postoperative recovery and complication rates. By focusing on these dynamics, this research aims to contribute to the development of more effective pre-surgical assessments and postoperative care strategies for this high-risk population.

In this study, a diverse cohort of patients was recruited at a level 1 trauma center over two years. The AUDIT-C questionnaire was used to categorize patients based on their alcohol consumption (18). While the AUDIT-C is a widely used tool for assessing alcohol use, it relies on self-reported data, which is subject to bias (29). Patients may underreport their alcohol consumption due to social desirability, or memory recall issues (30, 31). This limitation must be considered when interpreting the findings, as self-reported alcohol consumption may not accurately reflect actual intake levels. Despite its limitations, the AUDIT-C remains a valuable screening tool, but its results should be interpreted cautiously (18). Moreover, the AUDIT-C assessment provides a retrospective evaluation and may not adequately reflect the consequences of acute alcohol intoxication or withdrawal. Nevertheless, the aim of this study was to examine the impact of chronic alcohol consumption patterns and drinking behaviors on postoperative outcomes rather than to prioritize the effects of acute alcohol exposure.

To investigate the influence of alcohol on surgical outcomes, patients were also divided into control and complication groups. Interestingly, patients who consumed alcohol were, on average, younger and had lower BMIs than their non-drinking counterparts. However, the duration of hospital stays remained consistent across all groups. The relatively high average age observed in both groups can be attributed to the inclusion of elective arthroplasty patients within our cohort. Unlike acute trauma cases, elective joint replacement patients tend to be older due to the prevalence of degenerative joint diseases requiring surgical intervention. This inclusion influences the overall age distribution and must be considered when interpreting demographic differences between alcohol-consuming and non-consuming patients. This demographic trend underscores the complexity of the patient population, with age and BMI playing significant roles alongside alcohol consumption in influencing health outcomes (32, 33). This complexity further emphasizes the importance of matching patients based on relevant criteria, such as age, BMI, and other confounding factors. While this approach may result in a smaller sample size, it offers higher-quality data and more clinically relevant findings.

Both trauma severity and surgical intervention are known to have a relevant impact on the immune system. We acknowledge the importance of trauma severity in influencing immune responses. Although specific trauma severity scores (e.g., Injury Severity Score) were unavailable for this study, we categorized injuries based on anatomical site and documented surgical interventions. Our analysis confirmed that the distribution of injury sites and types of surgical interventions was comparable across the alcohol-positive and alcohol-negative groups. However, the absence of a formal trauma severity score is a limitation of this study. Future studies would benefit from incorporating detailed trauma severity scores (e.g., ISS) and matching patients accordingly to improve comparability.

Analyzing standard laboratory parameters, revealed that many parameters of the patients’ routine blood screening in the clinic did not show significant differences between the groups. However, CRP emerged as a notable exception. CRP is an acute-phase protein, produced by the liver in response to systemic inflammation (34). Trauma injuries often trigger an inflammatory response in the body, leading to elevated CRP levels (35). This increase was observed across all studied groups, reflecting the normal inflammatory reaction to trauma. However, CRP levels were significantly elevated in the alcohol-positive complication group compared to both the alcohol-negative and alcohol-positive control groups. This finding underscores the possible role of alcohol in exacerbating the inflammatory response, contributing to the development of complications. The ROC curve analysis on the other side, demonstrated an AUC of 0.6288 a suboptimal discriminatory ability as a predictive marker for complications within the alcohol-positive group. This is likely due to the influence of various factors on CRP levels, including injury severity and lifestyle characteristics (35, 36). However, CRP’s sensitivity to inflammation makes it a valuable tool for monitoring recovery and differentiating between groups experiencing varying levels of postoperative stress or infections (37–40).

The comprehensive cytokine and protein screening conducted in this study provides valuable insights into the inflammatory and immune responses of the patients. Many of the complications observed were linked to infection, impaired wound healing, and systemic inflammatory responses. Therefore, targeting cytokines that play a critical role in inflammation and immune modulation was prioritized to understand and predict complications. One of the key observations was the general trend of increased inflammatory markers in the alcohol-negative complication group compared to controls, while the alcohol-positive complication group exhibited a decrease in these markers. These differences highlight the complex and potentially suppressive effects of alcohol on the immune system (41). Chronic alcohol consumption is known to impair the immune response, leading to a dampened inflammatory reaction (42). This could explain the reduced levels of cytokines in the alcohol-positive group in response to trauma, which may, make it more difficult to define specific immune biomarkers before complications may occur, due to the already impaired inflammatory response.

Additionally, the decrease in T/B cell activation markers observed in both complication groups suggests a compromised adaptive immune response. This is particularly concerning as a robust immune response is critical for effective recovery post-surgery (43, 44). The reduction in these markers may reflect an impaired ability of the immune system to respond adequately to surgical stress and potential infections. Thereby, increasing the risk of postoperative complications (45). This finding aligns with existing literature that describes alcohol’s inhibitory effects on T and B cell function (8), further complicating the clinical management of alcohol-consuming patients.

The highlighted cytokines CD28, B7-1, Eotaxin-3, TIMP-1, and IL-13 demonstrated distinct differences across the groups, indicating their potential as biomarkers for predicting postoperative outcomes. CD28 and B7-1 are crucial co-stimulatory molecules involved in T-cell activation and immune regulation. Their altered expression suggests disrupted immune signaling in patients with complications (46, 47). IL-13 is a cytokine involved in various immune and inflammatory processes. It is a key mediator of tissue fibrosis, particularly in conditions such as hepatic fibrosis (48). Likewise, eotaxin-3 and TIMP-1 are associated with inflammatory processes and tissue remodeling, respectively (49, 50). Both immune modulators play a role in hepatic injury and systemic inflammatory responses. Chronic liver diseases are closely linked to bone metabolism disturbances, including reduced osteoblast activity and impaired mineralization, (51). Thus, these cytokines were prioritized in our study. Due to their role in immune regulation, fibrosis, and tissue remodeling, they are also of particular importance for alcohol-related complications as they contribute to liver dysfunction, which in turn impairs bone homeostasis (52). To gain deeper insights into the role of these cytokines, their serum concentrations were measured using ELISA. The results revealed distinct differences between the control and complication subgroups in the negative-alcohol group, suggesting their potential as biomarkers. However, in the alcohol-positive group, this distinction was lost. Indicating that alcohol appears to neutralize or alter the cytokine response, making it even more challenging to identify reliable biomarkers for complications in patients with a history of alcohol consumption.

One of the key areas of concern regarding alcohol consumption is its impact on liver function (53). Chronic alcohol consumption is known to impair liver function, leading to dysregulation of immune responses and inflammation (54–56). This potential liver injury may additionally contribute to the altered expression and production of cytokines observed in the alcohol-positive groups (42). Hence, identifying a reliable biomarker for alcohol-related complications is particularly challenging due to the complex and multifactorial nature of alcohol’s impact on the body. Alcohol consumption can modulate immune responses, impair liver function, and exacerbate inflammatory processes, all of which can interfere with potential biomarker signals. This complexity makes it difficult to find a single indicator that consistently predicts complications, especially in clinical settings where patients exhibit varying levels of alcohol exposure and diverse health statuses. The interplay of these factors underscores the need for comprehensive research to develop effective biomarkers for predicting alcohol-related complications.

In conclusion, alcohol consumption not only complicates patient outcomes but also poses challenges for identifying reliable biomarkers of complications. The neutralization of cytokine responses in alcohol-positive patients underscores the need for further research into alternative pathways and markers that may better predict complications in this population.

## Data Availability

The original contributions presented in the study are included in the article/**Supplementary Material**. Further inquiries can be directed to the corresponding author.

## Supplementary material

Supplementary Table 1 Frequency of admission diagnoses in patients classified negative or positive for alcohol risk. This table presents the distribution of admission diagnoses among patients classified as negative or positive for alcohol risk in the study. The 20 most common diagnoses are listed, highlighting their frequency within each group. This comparison provides insight into potential differences in disease patterns between the two populations.

Supplementary Table 2 Frequency of surgery procedures in patients classified negative or positive for alcohol risk The table presents the 20 most common surgical procedures identified in the overall patient population of our study. These surgeries are analyzed based on patients classified as negative or positive for alcohol risk.
