# Supplementary material for: Impact of alcohol consumption on outcomes and potential of immune biomarkers for postoperative complications in trauma patients
Source: Front Immunol. 2025 Apr 14;16:1492288. doi: 10.3389/fimmu.2025.1492288 (PMC12034740; doi:10.3389/fimmu.2025.1492288)
Supplement: Supplementary Table 1 — Frequency of admission diagnoses in patients classified negative or positive for alcohol risk. This table presents the distribution of admission diagnoses among patients classified as negative or positive for alcohol risk in the study. The 20 most common diagnoses are listed, highlighting their frequency within each group. This comparison provides insight into potential differences in disease patterns between the two populations. [file Table1.docx]

Supplementary Tables:

**Supplementary** **Table 1: Frequency of admission diagnoses in patients classified negative or positive for alcohol risk**

|  | | Negative for Alcohol Risk | | Positive for Alcohol Risk | |
| --- | --- | --- | --- | --- | --- |
| TOP 20 Diagnose over all | ICD-10 Code | Control | Complication | Control | Complication |
| Hip Osteoarthritis | M16 | 108 (21.8%) | 8 (8.2%) | 49 (18.7%) | 0 (0%) |
| Gonarthrosis | M17 | 101 (20.4%) | 10 (10.2%) | 43 (16.4%) | 5 (11.4%) |
| Fractures of the Femur | S82 | 66 (13.3%) | 12 (12.2%) | 48 (18.3%) | 10 (22.7%) |
| Fractures of the Shoulder Girdle and Upper Arm | S42 | 44 (8.9%) | 6 (6.1%) | 24 (9.2%) | 4 (9.1%) |
| Femoral Neck Fracture | S72 | 45 (9.1%) | 2 (2%) | 22 (8.4%) | 0 (0%) |
| Fractures of the Forearm | S52 | 31 (6.3%) | 7 (7.1%) | 12 (4.6%) | 4 (9.1%) |
| Infection of a Prosthesis or Implant | T84 | 15 (3%) | 10 (10.2%) | 5 (2%) | 7 (15.9%) |
| Fractures of the Foot | S92 | 18 (3.6%) | 2 (2%) | 13 (5%) | 3 (6.8%) |
| Fractures of the Lumbar Spine | S32 | 10 (2%) | 2 (2%) | 10 (3.8%) | 3 (6.8%) |
| Pathological Fracture Due to Osteoporosis | M84 | 5 (1%) | 4 (4.1%) | 2 (0.8%) | 0 (0%) |
| Complications Following a Surgical Procedure (e.g., Postoperative Infections) | T81 | 1 (0.2%) | 4 (4.1%) | 2 (0.8%) | 1 (2.3%) |
| Other Osteoarthritis | M19 | 2 (0.4%) | 1 (1%) | 3 (1.1%) | 0 (0%) |
| Osteonecrosis (Bone Necrosis) | M87 | 4 (0.8%) | 0 (0%) | 2 (0.8%) | 0 (0%) |
| Fractures of the Femur | S86 | 2 (0.4%) | 1 (1%) | 2 (0.8%) | 1 (2.3%) |
| Fractures of the Ankle | S93 | 4 (0.8%) | 1 (1%) | 1 (0.4%) | 0 (0%) |
| Deforming Osteoarthritis | M21 | - | 3 (3.1%) | 2 (0.8%) | 0 (0%) |
| Osteomyelitis (Bone Infection) | M86 | 2 (0.4%) | 3 (3.1%) | 0 (0%) | 0 (0%) |
| Other Skin Disorders | L98 | 1 (0.2%) | 2 (2%) | 0 (0%) | 1 (2.3%) |
| Fractures of the Thoracic Spine | S22 | 3 (0.6%) | 0 (0%) | 1 (0.4%) | 0 (0%) |
| Injuries to the Hip and Thigh | S76 | 3 (0.6%) | 1 (1%) | 0 (0%) | 0 (0%) |

This table presents the distribution of admission diagnoses among patients classified as negative or positive for alcohol risk in the study. The 20 most common diagnoses are listed, highlighting their frequency within each group. This comparison provides insight into potential differences in disease patterns between the two populations.

Supplementary Table 2. Frequency of surgery procedures in patients classified negative or positive for alcohol risk

|  |  | Negative for Alcohol Risk | | Positive for Alcohol Risk | |
| --- | --- | --- | --- | --- | --- |
|  |  | Control | Complication | Control | Complication |
| Top 20 Procedures over all | OPS Code |  |  |  |  |
| Hip joint replacement | 5-820 | 123 (10.7%) | 7 (0.6%) | 59 (5.1%) | 1 (0.1%) |
| Knee joint replacement | 5-822 | 93 (8.1%) | 6 (0.5%) | 38 (3.3%) | 3 (0.3%) |
| Open reduction of a multi-fragment fracture in the joint area of ​​a long bone | 5-794 | 73 (6.4%) | 16 (1.4%) | 38 (3.3%) | 8 (0.7%) |
| Closed reduction of a fracture or epiphyseal separation with osteosynthesis | 5-790 | 45 (3.9%) | 4 (0.3%) | 21 (1.8%) | 2 (0.2%) |
| Open reduction of a simple fracture in the joint area of ​​a long bone | 5-793 | 25 (2.2%) | 3 (0.3%) | 18 (1.6%) | 2 (0.2%) |
| Operations on other bones: removal of osteosynthesis material | 5-787 | 23 (2%) | 4 (0.3%) | 8 (0.7%) | 2 (0.2%) |
| Open reduction of a multi-fragment fracture of small bones | 5-796 | 10 (0.9%) | 2 (0.2%) | 7 (0.6%) | 2 (0.2%) |
| Open reduction of a fracture of the talus and calcaneus | 5-797 | 11 (1%) | 1 (0.1%) | 6 (0.5%) | 1 (0.1%) |
| Endoprosthetic joint and bone replacement: Other joint plastic surgery | 5-829 | 6 (0.5%) | 1 (0.1%) | 7 (0.6%) | 1 (0.1%) |
| Open reduction of a multi-fragment fracture in the shaft region of a long bone | 5-792 | 7 (0.6%) | 2 (0.2%) | 4 (0.3%) | 0 (0%) |
| Pain Therapy: Complex Acute Pain Treatment | 8-919 | 5 (0.4%) | 4 (0.3%) | 1 (0.1%) | 2 (0.2%) |
| Open reduction of an acetabular and femoral head fracture with osteosynthesis | 5-799 | 4 (0.3%) | 1 (0.1%) | 4 (0.3%) | 2 (0.2%) |
| Open surgical arthrodesis | 5-808 | 4 (0.3%) | 5 (0.4%) | 2 (0.2%) | 0 (0%) |
| Operations on the skin and subcutaneous tissue: Surgical wound debridement | 5-896 | 1 (0.1%) | 4 (0.3%) | 4 (0.3%) | 2 (0.2%) |
| Operations on muscles, tendons, fascia and bursae. | 5-855 | 5 (0.4%) | 2 (0.2%) | 2 (0.2%) | 1 (0.1%) |
| Closed reduction of a joint dislocation with osteosynthesis | 5-79a | 4 (0.3%) | 1 (0.1%) | 4 (0.3%) | 0 (0%) |
| Shoulder Joint replacement | 5-824 | 5 (0.4%) | 0 (0%) | 0 (0%) | 3 (0.3%) |
| Open reduction of a simple fracture of small bones | 5-795 | 4 (0.3%) | 0 (0%) | 3 (0.3%) | 0 (0%) |
| Revision, replacement and removal of a hip joint endoprosthesis | 5-821 | 4 (0.3%) | 2 (0.2%) | 0 (0%) | 1 (0.1%) |
| Operations on other bones: osteosynthesis procedures | 5-786 | 2 (0.2%) | 1 (0.1%) | 3 (0.3%) | 0 (0%) |

The table presents the 20 most common surgical procedures identified in the overall patient population of our study. These surgeries are analyzed based on patients classified as negative or positive for alcohol risk.
